# Categorising specimen referral delays for CD4 testing: How inter-laboratory distances and travel times impact turn-around time across a national laboratory service in South Africa

**DOI:** 10.4102/ajlm.v9i1.1120

**Published:** 2020-12-21

**Authors:** Naseem Cassim, Lindi M. Coetzee, Deborah K. Glencross

**Affiliations:** 1 National Health Laboratory Service, Johannesburg, South Africa; 2 Department of Molecular Medicine and Haematology, Faculty of Health Sciences, University of the Witwatersrand, Johannesburg, South Africa

**Keywords:** HIV, cluster of differentiation 4, CD4, immune status, inter-laboratory referral, distance, travel time

## Abstract

**Background:**

The South African National Health Laboratory Service provides laboratory services for public sector health facilities, utilising a tiered laboratory model to refer samples for CD4 testing from 255 source laboratories into 43 testing laboratories.

**Objective:**

The aim of this study was to determine the impact of distance on inter-laboratory referral time for public sector testing in South Africa in 2018.

**Methods:**

A retrospective cross-sectional study design analysed CD4 testing inter-laboratory turn-around time (TAT) data for 2018, that is laboratory-to-laboratory TAT from registration at the source to referral receipt at the testing laboratory. Google Maps was used to calculate inter-laboratory distances and travel times. Distances were categorised into four buckets, with the median and 75th percentile reported. Wilcoxon scores were used to assess significant differences in laboratory-to-laboratory TAT across the four distance categories.

**Results:**

CD4 referrals from off-site source laboratories comprised 49% (*n* = 1 390 510) of national reporting. A positively skewed distribution of laboratory-to-laboratory TAT was noted, with a median travel time of 11 h (interquartile range: 7–17), within the stipulated 12 h target. Inter-laboratory distance categories of less than 100 km, 101–200 km, 201–300 km and more than 300 km (*p* < 0.0001) had 75th percentiles of 8 h, 17 h, 14 h and 27 h.

**Conclusion:**

Variability in inter-laboratory TAT was noted for all inter-laboratory distances, especially those exceeding 300 km. The correlation between distance and laboratory-to-laboratory TAT suggests that interventions are required for distant laboratories.

## Introduction

Laboratory services in South Africa are provided within the public sector by the National Health Laboratory Service (NHLS).^1^ This mandate is delivered through a national network of 255 laboratories ranging from large high-throughput laboratories located in metropolitan areas to smaller laboratories placed in rural district hospitals.^1^ Testing is typically offered using a tiered laboratory service approach.^2^ In the lower tiers, a basket of eight standard pathology tests are performed locally to provide emergency services and include full blood count, urea and electrolytes, cardiac enzymes, glucose, liver enzymes, among others. Specialised assays and tests supporting local HIV/AIDS and tuberculosis testing regarded as high-volume workloads are transferred through the laboratory referral network to appropriate specialist and dedicated testing laboratories, depending on the nature of the test. Tertiary centres and academic laboratories have high workloads that are facilitated by using more sophisticated, high-throughput analysers.^2^ CD4 testing, for example, is centralised into 43 laboratories located strategically across the country to ensure widespread full-service coverage.^2,3^ In South Africa, samples are collected daily from health facilities through a network of couriers and transported to the local source laboratory. These laboratories perform a basic repertoire of tests. More specialised tests are referred to the nearest testing laboratory with an expanded test repertoire.

A CD4 count is used to assess the patient’s immune status (level of immune suppression) at presentation and during therapy.^2^ Additionally, it assesses the risk of opportunistic disease, including co-infections such as cryptococcal meningitis in patients with CD4 counts lower than 100 cells/*µ*L.^4,5^ Global estimates of advanced HIV disease have remained largely unchanged over the last 5 years despite increased antiretroviral therapy (ART) coverage in low-and middle-income countries.^6^ Between 30% and 50% of HIV-positive individuals in low- and middle-income countries are estimated to have CD4 counts under 200 cells/*µ*L.^6^ In South Africa, Coetzee et al. reported that the percentage of samples with a CD4 count under 100 cells/*µ*L ranged between 7.8% and 11.5% for the 2014/2015 financial period across South Africa’s nine provinces,^7^ while the burden of advanced disease (CD4 count ≤ 200 cells/*µ*L), reported by Carmona et al., approaches nearly 40% among those entering care for the first time in 2016.^8^ Approximately 3.5 million CD4 tests are typically processed per annum throughout the NHLS. The burden of advanced disease thus weighs heavily on this laboratory service. Patients with advanced HIV disease are expedited into care and ART initiation, usually within 7 days of diagnosis.^6^ To meet the requirements of ‘fast-tracking’ patients into care and onto ART, CD4 results need to be delivered timeously to referring health facilities. This, in turn, requires that samples be delivered in good time to the testing laboratories, within a 12-h window (as per internal guideline), to allow laboratory testing to commence and be completed within the organisational stipulated testing inter-laboratory turn-around time (TAT) of 40 h. Within the NHLS, there are 255 source laboratories that refer CD4 samples to the 43 testing laboratories (5.9:1). The organisational TAT cut-off for the inter-laboratory referral of CD4 samples (or ‘lab-to-lab’ time) is set at 12 h.

Previous work revealed that the placement of CD4 testing equipment and testing capacity, within an area identified with longer TAT attributable to pre-analytical causes, led to a marked shortening of the associated pre-analytical TAT and noticeably shorter overall TAT.^9^ Glencross et al. reported using a service radius around existing CD4 testing laboratories to determine healthcare facilities and clinics that lay outside of existing service precincts.^2^ This study facilitated the identification of additional testing sites required to improve local TAT.^2^

To achieve prompt sample referral within the CD4 network to address pre-analytic TAT, the NHLS employs a ‘hub and spoke’ approach whereby each testing CD4 laboratory receives referred samples from multiple source laboratories where the samples are first accepted into the laboratory network (but where there are no facilities to perform the testing).^10^ At the referring (source) laboratory, the CD4 sample is registered (as a referral) onto the laboratory information system (LIS). Thereafter, samples are transported to the centralised testing site; the inter-laboratory timeframe is completed when the samples are received and receipted on the LIS at the testing site. The LIS automatically generates the relevant dates and times of sample dispatch and receipt that are used to determine the inter-laboratory TAT in hours.^10^ For the purpose of this study, this inter-laboratory time period (component), from registration at the source laboratory to referral receipt at the testing laboratory, is termed the ‘lab-to-lab’ TAT.

It is anticipated that the distance and travel time for inter-laboratory referral could potentially impact lab-to-lab TAT performance. Laboratories located more than 300 km from a testing laboratory are expected to have longer lab-to-lab TAT. Conversely, laboratories within a 100 km radius are expected to have shorter travel times and lab-to-lab TAT. With a decentralised laboratory service model, it would be assumed that an inter-laboratory referral distance would not exceed 250 km. The 250 km radius is based on the study by Cassim et al. that used the integrated tiered service delivery model (ITSDM) coverage precinct approach to address ART-related testing service coverage gaps.^2,10^ The Cassim et al. study assumed that a 250 km buffer around each laboratory would adequately address ART-related test coverage.^10^ The relationship between travel time, distance and lab-to-lab TAT performance would identify gaps in the current service model, where additional decentralisation would deliver better inter-laboratory TAT performance.

The objective of this study was to determine the impact of distance on inter-laboratory referral time in 2018 for public sector testing in South Africa.

## Methodology

### Ethical considerations

Ethical clearance was obtained from the University of the Witwatersrand (M1706108). This study was a retrospective analysis of NHLS laboratory data, did not use any patient identifiers and did not involve any direct patient contact.

### Study design and data analysis

This study used a cross-sectional study design. Microsoft Access and Excel (Microsoft Corp., Redmond, California, United States) were used to prepare data^11^ and analysis was performed using SAS 9.4 (SAS, Cary, North Carolina, United States).^12^ Laboratory data for all CD4 samples referred for centralised testing were extracted from the corporate data warehouse for the 2018 calendar year across South Africa. The corporate data warehouse is an entity of the NHLS and houses all LIS data generated by its laboratories. A referral CD4 outcome was assigned where both the source (referring) laboratory and testing laboratories fields were populated in the LIS data. Google Maps (Alphabet, Inc., Mountain View, California, United States) was used to calculate both inter-laboratory distances and travel times using confirmed coordinates of laboratories (latitude and longitude).^13^ Data were summarised in a worksheet for later analysis. The LIS date and time stamps were used to calculate the lab-to-lab (inter-laboratory referral) time in hours (time from registration at the source laboratory to the time of referral receipt at the testing laboratory). Due to the limited data points, the following delays could not be determined and are accounted for in the lab-to-lab TAT: (1) between registration at the source laboratory to pick up by the courier, (2) transport to the testing laboratory and (3) delivery at the testing laboratory for referral receipt.

Skewness can be quantified as a representation of the extent to which a given distribution varies from a normal distribution.^14^ A normal distribution reports a skewness of zero compared to a positively skewed distribution with a value of greater than zero.^14^ For a normal distribution, descriptive statistics such as the mean and standard deviation can be reported. However, for a skewed distribution, the median and interquartile range are reported. Hence, skewness dictates what descriptive statistics may be reported.

For each CD4 sample, the requesting health facility is captured to facilitate the delivery of patient reports. In the LIS, each health facility has a designated location code. This code is captured in the LIS based on health facility details provided on the laboratory request form. Unfortunately, very few health facilities had latitude and longitude data making it difficult to calculate the distance to the local source laboratory.

CD4 samples that were not referred (i.e. that were received directly at testing laboratories) were excluded for the purpose of this study. Data were reported as descriptive statistics, and visualised using histograms. A scatter plot was used to demonstrate the relationship between referral times (hours) and distances (km), with descriptive statistics reported. The inter-laboratory distances between source and testing sites were categorised into four buckets; (1) ≤ 100 km, (2) 101–200 km (3) 201–300 km and (4) > 300 km. The aim of the analysis was to determine whether lab-to-lab TAT was influenced by distance. The analysis could also reveal whether decentralisation within a 200 km radius would decrease lab-to-lab TAT. The number of health facilities associated with local source laboratories in each of the four inter-laboratory distance buckets was reported. The lab-to-lab TAT component and descriptive statistics (median and 75th percentile) were reported, using the non-parametric Wilcoxon scores (rank sum) test to determine differences in TAT reported among the distance categories. In addition, inter-laboratory referral routes longer than 300 km that referred 1000 or more samples in 2018 are reported as a separate table reporting: (1) source laboratory, (2) testing laboratory, (3) distance, (4) travel time (hours), (5) percentage within 12-h cut-off and (6) 75th percentile, using source and testing laboratory aliases. The projected daily referral volumes were calculated based on the assumption of 21.73 working days per month.

## Results

There were 2 844 242 CD4 samples performed in 2018, of which 1 390 510 (49%) were referred for centralised testing within the NHLS laboratory network.

### Distribution of inter-laboratory referral times and distances

**FIGURE 1:**
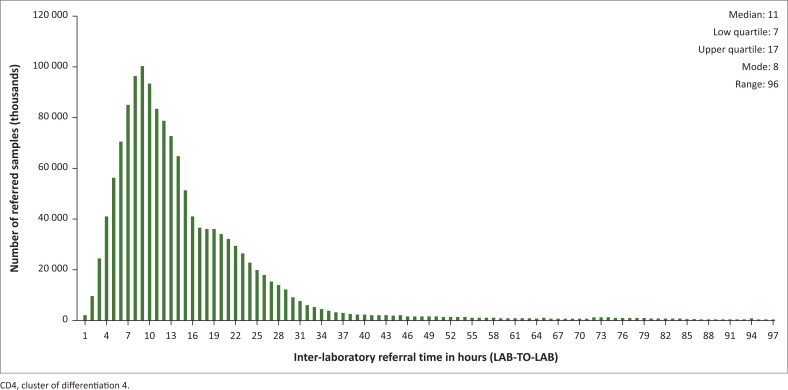
Distribution of inter-laboratory CD4 testing referral laboratory-to-laboratory turn-around time in the 2018 calendar year for National Health Laboratory Service laboratories in South Africa. Median, interquartile range, mode and range are indicated (inset).

A linear relationship exists between referral times and distances. lab-to-lab data reported a positively skewed distribution (skewness = 2.83). A lab-to-lab median of 11 h with an interquartile range of 7–17 h was reported. The mode was 8 h, with a range of 96 h (Figure 1).

**FIGURE 2:**
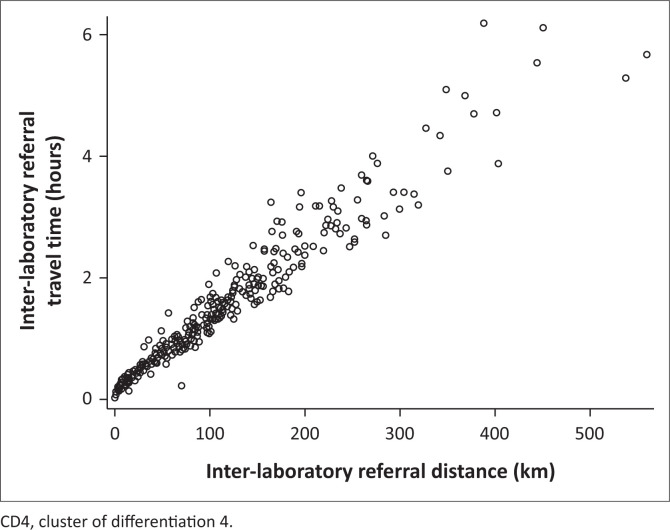
Relationship between inter-laboratory CD4 testing referral distance and travel time between source and CD4 testing laboratories in the 2018 calendar year across the National Health Laboratory Services in South Africa.

The inter-laboratory CD4 testing referral distance data also reported a positively skewed distribution (skewness = 1.66) (Figure 2). The median distance travelled by referred samples was 67.1 km (interquartile range = 20.1–123.0 km; mode = 14.4 km; range = 559 km). The distribution of inter-laboratory referral travel times was also positively skewed (skewness = 1.33). The median was 0.9 h, with an interquartile range of 0.4–1.63 h, a mode of 0.35 h, and a range of 6.15 h.

### Inter-laboratory CD4 testing referral performance by distance category

Overall, 67% of samples were referred to testing laboratories within a 100 km inter-laboratory referral distance, increasing to approximately 93% of samples within a 200 km radial precinct. Approximately 7% of samples were transported 201 km or more from their source laboratory to their sister centralised testing laboratory. Overall, for referral distances under 300 km, all inter-laboratory precincts (both the mode and median lab-to-lab TAT) met the organisational inter-laboratory TAT cut-off of 12 h.

Longer lab-to-lab TATs were noted from referral or source laboratories further than 300 km from testing laboratories. Analysis of the 75th percentile lab-to-lab TAT, unlike the mode and median parameters, revealed that only inter-laboratory referral distances of under 100 km met the 12-h cut-off (Table 1). The Wilcoxon scores (rank sum) test indicated a significant difference in lab-to-lab TAT across the four distance categories.

**TABLE 1:** Inter-laboratory CD4 testing referral laboratory-to-laboratory performance by distance categories in the 2018 calendar year for National Health Laboratory Services laboratories in South Africa.

Distance category†	Number of health facilities‡		Number of referred samples (%)§		Number of source laboratories (%)		Cut-off	Mode	Median	75th percentile
	*n*	%	*n*	%	*n*	%				
≤ 100 km	2169	45.7	936 667	67.4	124	49	12	7	11	8
101–200 km	1644	34.6	356 162	25.6	86	34	12	8	11	17
201–300 km	576	12.1	73 596	5.3	29	11	12	9	10	14
> 300 km	357	7.5	23 815	1.7	16	6	12	12	15	27

Note: Wilcoxon rank sum test – *p* = < 0.0001.

CD4, cluster of differentiation 4.

† , Indicates the distance between the CD4 source laboratory that receives samples from clinics via a courier network and the testing laboratory. The further the distance, the higher the potential for an extended turnaround time affecting service delivery.

‡ , Number of laboratory information system location codes.

§ , Percentage of samples referred.

Laboratories serviced the majority of health facilities (80%) with an inter-laboratory referral distance within 200 km. There were 12% and 7.5% of health facilities that were serviced by laboratories with an inter-laboratory referral distance of 201 km – 300 km and more than 300 km.

#### Distant inter-laboratory routes performance analysis

For five source laboratories, distance to the nearest testing facility exceeded 300 km (Table 2). Distances ranged from 304 km to 560 km (mean distance of 387 km). The percentage of samples transferred to the centralised testing site within the cut-off TAT of 12 h was 36%, 37% 6%, 83% and 8% for source laboratories S1, S2, S3, S4 and S5. The median lab-to-lab TAT across each of the five sites exceeded the stipulated TAT cut-off for all five sites. The 75th percentile lab-to-lab TAT revealed further variation of pre-analytical inter-laboratory TAT efficiency, that is, 10 h – 29 h for individual laboratories. Daily CD4 referral volumes from these laboratories ranged from 6 to 24 samples.

**TABLE 2:** Laboratotry-to-laboratory performance of inter-laboratory referral routes over 300 km and involving at least 1000 referred samples in the 2018 calendar year for National Health Laboratory Service laboratories in South Africa.

Source laboratory†	Testing laboratory‡	Number of samples referred	Projected daily referrals§	Distance (km)¶	% within lab-to-lab cut-off	75th percentile lab-to-lab
S1	T1	6328	24	402	36	17
S2	T1	6312	24	350	37	15
S3	T2	4903	19	319	6	36
S4	T3	2378	9	304	83	10
S5	T4	1622	6	560	8	29

CD4, cluster of differentiation 4.

† , Laboratory that receives CD4 samples from health facilities that is delivered by a courier.

‡ , Laboratory that performs the CD4 test.

§ , Number of samples referred from a source to a testing laboratory.

¶ , Distance in kilometres between a source and a testing laboratory (inter-laboratory referral).

## Discussion

In this study, the impact of distance and travel time on lab-to-lab TAT as a pre-analytical component of total TAT was assessed to categorise laboratory service efficiency and, in national service, understand where bottlenecks of logistics may occur. We observed moderate correlation between distance travelled and the overall recorded inter-laboratory lab-to-lab TAT. However, across all service precincts, especially those exceeding a radius of 300 km, variable inter-laboratory TAT was recorded. Generally, median lab-to-lab TAT of referrals within a 200 km radius of a testing facility (*n* = 210 laboratories; 93% of total referrals) was 11 h and fell within the organisational stipulated inter-laboratory TAT cut-off of 12 h. This finding confirms that previous implementation of decentralised CD4 testing facilities into lower service tiers has positively impacted the efficiency of local service delivery.^2,9^ Earlier work identified that well-planned decentralisation in remote areas has been successfully implemented in the Northern Cape (De Aar, Upington and Tshwaragano laboratories).^2^

Despite previous decentralisation drives and a national median inter-laboratory TAT of 11 h for at least 93% of referred samples, there are still outliers noted. For example, where distances travelled to a centralised testing facility was between 100 km and 200 km, a 75th percentile inter-laboratory TAT of 17 h was noted. Therefore, although most of the referring times reported here were within the inter-laboratory transfer cut-off of 12 h, service gaps are evident (where referral laboratories failed to deliver their samples to the testing laboratory within the stipulated time frame).

Glencross et al. developed the ITSDM that consists of five tiers to facilitate sustainable ‘full-service coverage’ across South Africa.^2^ This model could be used to extend CD4 services into rural and remote areas with tier 3 (community) or tier 2 (point-of-care and hub) services that complement more centralised testing offered by tiers 1 and 2.^2^ The tier 3 sites would be installed in existing laboratories with existing infrastructure.^2^ Candidate laboratories are identified using the ITSDM,^2^ to service health facilities outside the current service precinct.^2,9^ The implementation of new tier 3 community laboratories in this context has a proven role in reducing pre-analytical TAT, particularly where samples are required to travel distances in excess of 300 km.^9^ Coetzee et al. have previously reported the successful impact of the placement of an ITSDM-identified community CD4 laboratory in an area experiencing long overall TAT; a dramatically reduced TAT was realised in this rural district.^9^ This was achieved by adding CD4 testing capacity to the existing test repertoire in the local laboratory (De Aar) which substantially reduced the pre-analytical component of total TAT of testing in the area. In the current study, based on the number of samples referred and the proportion of samples within cut-off TAT, sites 1 and 2 should be eligible for local capacity placement. In some cases (i.e. sites 3, 4 and 5), review of local logistics could improve the pre-analytical component, especially as it was revealed that one of the five sites, which was 304 km from its sister testing site, achieved a remarkable 75th percentile lab-to-lab TAT of around 10 h. This suggests that distance alone is not the primary determining factor influencing lab-to-lab TAT.

This study demonstrated a median lab-to-lab TAT of 15 h, with a right skewed distribution and a 75th percentile of 27 h for referrals outside a 300 km service precinct to the nearest testing laboratory. This finding, again, reiterates the need to apply the ITSDM approach described above to assist with identifying sites for capacity development and alleviation of the longer inter-laboratory TATs identified in this work. At least four sites with low test volumes and exceeding 300 km to the nearest testing facility warrant the introduction of a local service that could be accomplished with lower throughput platforms (daily capacity of 40 samples).^15,16,17^ Testing systems such as the Beckman Coulter Aquios cytometer or Becton Dickinson FACSPresto are easy to use and suitable for existing smaller laboratories requiring minimal refurbishment. Costing assessments done by Cassim et al. have demonstrated that the incremental cost for decentralising CD4 testing to community laboratories is a mere $2.05.^18^ This low incremental cost is due to the placement agreement model where the cost of the analysers for new sites is included in the reagent costs. Whether this is a high or low volume site requiring different platforms, the cost to offer one test remains the same. Furthermore, the proposed assays have been validated locally and have World Health Organization pre-qualification,^19^ reporting acceptable precision to predicate methods for both venous or capillary blood sampling.^15^ The challenges for the decentralisation model include the availability of adequately trained staff, the need to increase resupply points of reagents and consumables at lower-level facilities, and the ease of access to suppliers for maintenance and repairs. Irrespective of costs, the impact on patient care is invaluable.^20^

With only about 50% of tested CD4 samples referred between laboratories, the NHLS strives to have adequate testing facilities for maximum coverage especially in high-burden and remote areas,^2^ and, as mentioned above, has set a maximum target time of 12 h for samples to be transported from source laboratories to the centralised testing facilities. It would make sense for inter-laboratory TAT (referral time) to be directly proportional to distance. This assumption is based on the provision of adequate and reliable delivery vehicles and appropriately maintained roads. The road conditions and how the collection and transportation routes are designed would also affect TAT performance. The operating times of referral sites may also impact delays in TAT as not all offer a 24-h service. Human resource factors can also lead to delays and longer TAT, including the work hours, work ethos and diligence of both drivers and receiving office staff at the centralised facility.^21^

A limitation of this analysis is that it focuses on the inter-laboratory referral after samples are received within the laboratory network. The laboratory pre-analytical phase of TAT is measured from the first registration in an NHLS laboratory to receipt in the testing facility; it does not include the time from venepuncture to first registration on the LIS system. However, as the latter time periods are poorly recorded, it is difficult to determine if a longer lab-to-lab TAT is due to a delay in time from sample collection to registration at the source laboratory. Challenges in the pre-analytical and analytical arms would have to be addressed separately due to differences in the nature of challenges and the involvement of different personnel. Therefore, separate corrective action interventions need to be set in motion. This may include redesigning the specimen referral network which could include additional routes or potentially identifying some sites for point-to-point routing instead of a multi-stop route, or vice versa.^2^

To adequately address all delays of inter-laboratory referral TAT, all aspects of this phase should be addressed. Not all of the processes undertaken during the transfer of samples are documented and traceable, that is, not all steps generate a time and date stamp on the LIS or are date-stamped at the point of venesection. The introduction of electronic monitoring of all total TAT would facilitate a more complete view of possible points of delay across the entire sample journey and identify aspects for corrective action. Vertical audits can be used to identify sources of delay on different days of the week to calculate average (delay) times for each process.^22^ In addition, workflow analyses could assist in mapping out (and eliminating) processes or steps that do not add value.^22^ Through this process, a standard operating procedure for all pre-analytical processes could be introduced across facilities.

### Limitations

The study used predominantly laboratory data to assess the performance of CD4 test referrals across the NHLS. This study did not report data for other tests as the inter-laboratory referral network is integrated. All the concepts reported in this study for CD4 testing apply to other referred tests as well. No information was available to review time from sample collection to registration onto the laboratory network, as well as time after sample registration at the source laboratory to collection by the courier.

### Conclusion

This study demonstrates that most referrals for CD4 testing reach their testing facilities within the expected 12-h window, with some outliers identified. Here, differences in inter-laboratory referral distances and lab-to-lab TAT performance suggest that there are inconsistent systems and practices in use to transfer samples between centres. Further investigation to understand the root causes would assist in aligning efficient delivery of all samples between facilities. There is a need for additional data collection for the inter-laboratory referral process to better understand where service bottlenecks exist. This study identified the need for electronic data recording at multiple stages of sample inter-laboratory referral such that bottlenecks can easily be identified and resolved to optimise timely referrals. For distances exceeding 300 km, the establishment of additional community CD4 laboratories is recommended.
